# Terrestrial Vegetation Drives Methane Production in the Sediments of two German Reservoirs

**DOI:** 10.1038/s41598-019-52288-1

**Published:** 2019-11-04

**Authors:** Jörg Tittel, Matthias Hüls, Matthias Koschorreck

**Affiliations:** 1 Helmholtz Centre for Environmental Research - UFZ, Department Lake Research, Brückstraße 3a, D-39114 Magdeburg, Germany; 2 Leibniz-Laboratory for Radiometric Dating and Stable Isotope Research, Max-Eyth-Str. 11-13, D-24118 Kiel, Germany

**Keywords:** Limnology, Carbon cycle

## Abstract

Inland waters and reservoirs in particular are significant sources of methane to the atmosphere. However, little information is available on the extent to which organic carbon from terrestrial vegetation or from internal photosynthesis fuels the methane production. This limits our ability to constrain methane emissions efficiently. We studied the isotopic composition (^13^C, ^14^C) of pelagic and sedimentary carbon sources in two small German reservoirs. The methane was enriched by radiocarbon with isotopic ranges (∆^14^C 5‰ to 31‰) near to fresh terrestrial organic carbon (OC, 17‰ to 26‰). In contrast, potential source OC produced by internal photosynthesis was characterized by negative ∆^14^C values (−30‰ and −25‰) as derived from signatures of inorganic carbon in the reservoirs. The particulate OC in stream supplies (terrestrial OC) was also ^14^C depleted in almost all cases, but highly variable in ∆^14^C (−131‰ to 42‰). Although the import of terrestrial OC was lower than the amount of OC produced by reservoir-internal photosynthesis, we conclude that the methane production was predominantly fuelled by catchment vegetation. The utilized terrestrial OC was of contemporary origin, fixed within years to decades before sampling and supplemented with reservoir-internal or aged terrestrial OC. Our results indicate that terrestrial biomass is an important driver of methane production in reservoirs receiving significant imports of terrestrial OC.

## Introduction

Production of methane in lakes and reservoirs is an important process in the global carbon cycle^1^. Although carbon dioxide (CO_2_) emissions of surface waters are sustained by catchment soil respiration^2^, methane released from reservoirs and lakes is generated in these systems itself. The methane is mainly produced at anoxic sites, mostly in the sediments, as a product of the mineralization of organic matter. Methanogenesis is the thermodynamically least efficient pathway of organic matter mineralization and usually starts, when other electron acceptors (oxygen, nitrate, iron, sulphate) are depleted.

There are two pathways of methane production: acetate fermentation and CO_2_ reduction. In freshwater systems fermentation is the dominant process^3^. It depends on the supply of available organic carbon (OC)^4^. There are two sources of OC in lakes and reservoirs: (a) input of dissolved organic carbon (DOC) and particulate organic carbon (POC) from terrestrial sources of the catchment, and (b) autochthonous (internal) production by photosynthetic organisms in the water body itself. Here, we use the term ‘autochthonous’ with respect to the reservoir while ‘terrestrial’ refers to carbon imports by streams. While many studies have examined how much these sources contribute to internal carbon cycling^5–7^ and whether the primary substrate of methanogenic bacteria is acetate or H_2_ plus CO_2_^3^, less is known about the significance of autochthonous versus terrestrial OC as substrates for methane production. However, cost-effective measures in order to limit methane emissions should essentially focus on the predominant OC source. A better knowledge of the specific sources of methane would help choosing a purposeful strategy to reduce methane emissions from reservoirs.

Organic matter is a mixture of substances with varying degradability. If exposed to suitable conditions, easily biodegradable compounds are preferably mineralized by microbes. Autochthonous OC mostly originates from planktonic photosynthesis and was found to have a high biolability^8^. Recent studies show a positive correlation between trophic state and methane emissions suggesting that autochthonous carbon is a relevant carbon source for methane production in reservoirs’ sediments^9,10^. Terrestrial OC derives from plants and soils in the catchment. Unlike autochthonous material, it is often older and thus, already partly degraded. As a result, terrestrial OC tends to be more biorefractory, i.e. it is less available for microorganisms compared to autochthonous material^11,12^ and sediments receiving high inputs of terrestrial OC tend to have higher OC burial efficiencies^13^.

Terrestrial OC can be either dissolved or particulate, and it is the dominant fraction of organic matter in nutrient poor lakes^14^. In contrast to lakes, in reservoirs, terrestrial OC originates from two sources: (a) soil and plant material remaining from the time before reservoir construction and (b) input during reservoir filling and operation. In tropical reservoirs the degradation of terrestrial material originating from pre-impoundment times is the dominant carbon source of methane production during the first years of reservoir operation^15,16^. Reservoirs are known to be aquatic methane emission hotspots because they trap organic material originating from the catchment^17,18^. However, the close link between sedimentary methane production and terrestrial carbon input has been established from indirect evidence, like carbon budgets or correlations between methane emission and sediment quantity and composition. Direct evidence showing that sedimentary methane originates from terrestrial carbon sources is scarce. Experiments in which sediment samples were amended with different types of organic carbon verified terrestrial carbon as an important substrate for methane production in sediments^19^. The analysis of the isotopic composition of different carbon species in the environment offers the possibility to directly link substrates with reaction products *in situ*^20^. Unfortunately, the results of studies using exclusively the stable isotope ^13^C were not unambiguous. There was no relation between the δ^13^C of sediment organic matter and methane in 32 European lakes^21^.

Radiocarbon is a powerful but underused tool in geochemical studies. Compared to the stable isotope ^13^C, the natural abundances of the isotope ^14^C vary dynamically over a much broader range, which increases the probability that different sources can be distinguished. Radiocarbon in methane (radiomethane), in particular, has been analysed to explore the significance of old vs. recent carbon sources for methane production in various environments^22^. However, there are only a few radiomethane studies from lakes or reservoirs. Methane older than 20,000 years was found in lake Kivu, an African rift lake, where the methane was predominantly formed from geogenic sources of CO_2_ and H_2_^23^. The sediment of a reservoir in the northern boreal area of Finland contained methane that was produced from recently fixed or from older sources fixed 670 years before present (BP)^24^.

We used a multi-isotope approach to identify the carbon source of methane produced in the sediment of two drinking water reservoirs. We chose two reservoirs that were similar in size and catchment area but different in nutrient supply and trophic status. Due to the small size of the studied reservoirs the spatial gradients of OC supplied by streams are presumably less significant as in larger systems. By analysing the ^13^C and ^14^C content of the methane and of the potential carbon sources in the reservoirs and in the catchments we aimed to disentangle the role of autochthonous versus terrestrial sources for methane production.

## Materials and Methods

### Study sites and sampling

Rappbode and Hassel are adjacent reservoirs located upstream in a system of dams used for drinking water production. They are situated in the lower part of the Harz mountains, Germany (51.7092°N, 10.7981°E; 51.7091°N, 10.8319°E; respectively). Both reservoirs are small (Table 1) and with a total length of 1.6 to 1.8 km comparable in size to the river-reservoir transition zone of larger reservoirs. They receive inflows each from one stream but differ with respect to nutrient imports^25^. The Rappbode reservoir catchment is dominated by forest while there is some influence of agriculture on the more eutrophic Hassel reservoir^26,27^. Both bodies of water regularly develop an anoxic hypolimnion during summer stratification. At the time of sampling, dissolved oxygen was absent below 12 m depth. The thermocline was established at 9 m depth in Rappbode reservoir and at 7 m depth in Hassel reservoir. Both reservoirs showed only minor longitudinal gradients with respect to methane and CO_2_ concentrations, emissions and hydrochemistry. Plankton concentrations are typically increasing from the inflow to the dam^28^. Due to the mostly steep and rocky shore few aquatic macrophytes were only present at the inflow area. The sediment organic matter content ranged between 13 and 26% loss on ignition and tended to be highest at the deepest points^29^. The sampling stations were located at the deepest points 50 m away from the spillover. In Hassel reservoir, one additional sample was taken at a station near to the inflow at 4 m depth.

**Table 1:** Morphometry, trophic status and carbon fluxes of Hassel and Rappbode reservoirs.

	Rappbode reservoir	Hassel reservoir
Catchment area (km^2^)^*a^	47.6	44.6
Reservoir surface area (km^2^)^*a^	0.22	0.29
Mean depth (m) ^*a^	5.3	5.0
Maximum depth (m)^*a^	17	14
Residence time (days)	34	51
***Pools***		
Inflow total phosphorus (µg P L^−1^)^*b^	23	47
Chlorophyll *a* (µg L^−1^)^*c^	8.6	16.3
Phytoplankton biomass (g C m^−2^)^*d^	4.9	12.3
***Process rates***		
Internal photosynthesis (g C m^−2^ year^−1^)^*e^	46	130
DOC terrestrial import (g C m^−2^ year^−1^)^*f^	207	185
POC terrestrial import (POC-In) (g C m^−2^ year^−1^)^*g^	34	32
Benthic CH_4_ flux (g C m^−2^ year^−1^)^*h^	6	8
Benthic CO_2_ flux (g C m^−2^ year^−1^)^*h^	38	19
CH_4_ emission (g C m^−2^ year^−1^)^*i^	2.0	1.2

Residence time, carbon import and export values as well as stream total phosphorus (TP) refer to the sampling year (21.02.2012–19.02.2013).

^*a^Friese, *et al*.^25^.

^*b^Means of weekly samples of TP in inflowing streams Hassel and Rappbode, methods see Friese, *et al*.^25^.

^*c^Means of biweekly samples from 2 m depth, methods see Friese, *et al*.^25^.

^*d^Means of biweekly phytoplankton samples (18.01.12–10.12.12, 0–5 m depth, unpublished data, methods for phytoplankton biomass estimation see Friese, *et al*.^25^, a specific carbon content of 0.23 mg C mm^−3^ was assumed).

^*e^Estimated on the basis of phytoplankton biomass in 2012 (see above^*c^) and biomass-specific photosynthetic production. The latter was derived from net primary production (2013 in Hassel reservoir, 2014 in Rappbode reservoir; Morling, *et al*.^58^) and corresponding phytoplankton biomass during production measurements.

^*f^Tittel *et al*.^31^, therein reservoir imports were referred to as’yields’ with respect to catchment areas. Here, the imports were related to the reservoir surface area.

^*g^See POC-In of Table 3.

^*h^Flux of methane or CO_2_ out of the sediment into anoxic hypolimnetic water measured in 2011. Fluxes were estimated from accumulation rates in the anoxic hypolimnion during summer^44^.

^*i^Methane flux between reservoir and atmosphere measured seasonally in 2015 with floating chambers (Table S2).

Samples characterizing the total inorganic carbon (TIC) in the surface layer (TIC-Sur) as well as the outflow POC (POC-Out) were both obtained using a boat near the spillover from 0.5–1.0 m depth. The samples were collected weekly from 21.02.12 until 19.02.13. We used a Limnos water sampler (Turku, Finland) and 100 mL (TIC) and 200 mL (POC) acid-rinsed, brown glass flasks with solid ground glass stoppers. For ^13^C- and ^14^C-TIC isotopes, every second week we collected water samples of 200 mL and 1 L volume, respectively in acid-rinsed and baked (500 °C, 4 h) glass bottles. We added 0.2 mL of a HgCl_2_ solution (1%) to the ^13^C samples before they were closed with crimp caps (20 mm, aluminium and butyl/PTFE). For analysis of ^13^C- and ^14^C-POC-Out isotopes 1 L volume, acid rinsed and baked flasks were used. Table 2 gives an overview of parameters and sampling.

**Table 2:** Description of parameters used in this manuscript.

	Parameter description	Sampling frequency	Data source
***Catchment***			
∆^14^C-POC-Needles	^14^C isotope concentration in tree needles characterising fresh terrestrial^*^ OC	2 samples	^26^
∆^14^C-POC-Soil	^14^C isotope concentration in soil OC characterising aged terrestrial^*^ OC	3 samples	^26^
***Stream inflow (reservoir import)***			
POC-In	concentration or flux of terrestrial^*^ POC	weekly	^26^
δ^13^C-POC-In	^13^C isotope concentration in terrestrial^*^ POC	4 samplings	this study
∆^14^C-POC-In	^14^C isotope concentration in terrestrial^*^ POC	5–7 samplings	^26^
***Outflow (reservoir export)***			
POC-Out	POC concentration or flux	weekly	this study
δ^13^C-POC-Out	^13^C isotope concentration in POC, time-integrated^#^	every 2 weeks	this study
∆^14^C-POC-Out	^14^C isotope concentration in POC, time-integrated^#^	every 2 weeks	this study
***Surface layer of reservoir***			
TIC-Sur	TIC concentration	weekly	this study
δ^13^C-Sur	^13^C isotope concentration in TIC, time-integrated^#^	every 2 weeks	this study
∆^14^C-Sur	^14^C isotope concentration in TIC, time-integrated^#^, represents ∆^14^C of autochthonous^*^ OC	every 2 weeks	this study
δ^13^C-autumn	^13^C isotope concentration in TIC, end of summer stratification	single sample	this study
∆^14^C-autumn	^14^C isotope concentration in TIC, end of summer stratification, represents ∆^14^C of autochthonous^*^ OC	single sample	this study
***Sediment of reservoir***			
CO_2_-Sed	CO_2_ concentration relative to sediment volume	single sample	this study
δ^13^C-CO_2_-Sed	^13^C isotope concentration in CO_2_	single sample	this study
∆^14^C-CO_2_-Sed	^14^C isotope concentration in CO_2_	single sample	this study
POC-Sed	POC concentration relative to sediment volume	single sample	this study
δ^13^C-POC-Sed	^13^C isotope concentration in POC	single sample	this study
∆^14^C-POC-Sed	^14^C isotope concentration in POC	single sample	this study
CH_4_-Sed	CH_4_ concentration relative to sediment volume	single sample	this study
δ^2^H-CH_4_-Sed	^2^H isotope concentration in CH_4_	single sample	this study
δ^13^C-CH_4_-Sed	^13^C isotope concentration in CH_4_	single sample	this study
∆^14^C-CH_4_-Sed	^14^C isotope concentration in CH_4_	1–3 samples	this study

^#^See Methods.

^*^Terrestrial: import into the reservoir by streams, autochthonous: internal production in the reservoir by photosynthesis.

Sediment samples were taken on 17.10.2012 using a gravity corer (Uwitech, Mondsee, Austria). The first core (6 cm diameter, 10 cm depth) was used for sediment methane isotope (^2^H-CH_4_-Sed, ^13^C-CH_4_-Sed, ^14^C-CH_4_-Sed) and sediment CO_2_ stable carbon isotope (^13^C-CO_2_-Sed) analysis as well as for concentration measurement of both gases. In the boat the sediment was transferred into an ultra-high purity (UHP) nitrogen filled twist off jar of 1 L volume. We added 340 g sodium chloride to inhibit microbial activity^30^. The jar was immediately closed with a modified metal lid. The lid was equipped with two septa (butyl injection stoppers grey, 12 mm inner diameter, 18 mm outer diameter, produced for ND20 crimp vials) that were mounted before the sampling in two drilled holes to allow the sampling of gases (see below). The second core was taken for radiocarbon isotope measurement of CO_2_ (^14^C-CO_2_-Sed). The sediment but no sodium chloride was added to the nitrogen-filled jar before it was also closed with a modified lid. The third core (9 cm diameter, 10 cm depth) was extruded into zip plastic bags and used for POC isotope (^13^C- and ^14^C-POC-Sed) analysis and POC-Sed concentration measurement. Two further cores for ^14^C-CH_4_-Sed measurement were taken in Hassel reservoir, one from the deepest point and one from the shallow station near the inflow. In addition to Hassel and Rappbode, two other reservoirs were sampled once for ^14^C-TIC-Sur and ^14^C-CH_4_-Sed, i.e. Bautzen (Germany, 51.2175°N, 14.4665°E) and Sau (Spain, 41.9705°N, 2.3936E).

### Sample processing in the laboratory

The ^13^C and ^14^C TIC-Sur and TIC-autumn samples were processed as described earlier^31^. To collect CO_2_ for ^14^C analysis within 24 hours the ^14^C samples were acidified (HCl 37%, pH 2) and outstripped with UHP nitrogen for 4 hours. The inorganic carbon was precipitated as carbonates in a saturated and pre-filtered barium hydroxide solution. The precipitates were washed three times with nitrogen bubbled deionized water to remove the remaining barium hydroxide and then dried at 60 °C under continuous nitrogen supply in a throughflow system. The carbonates were stored in UHP nitrogen flushed vials closed by crimp caps (20 mm, aluminium and butyl/PTFE). To characterise the isotopic composition of TIC present in the surface layers during the annual cycle (TIC-Sur) the samples from a particular reservoir were combined to a bulk sample, weighed according to the yields (concentration times outflow) of TIC at individual sampling days. This applied to the dried carbonate precipitates, from which subsamples were weighed and then pooled for ^14^C analysis (^14^C-TIC-Sur) as well as to the mercury-stabilized ^13^C-TIC-Sur water samples, from which defined volumes were integrated to the bulk sample. Therefore, the analyses of our time-integrating samples are representative for the TIC near the dam that was exported from the reservoir during the sampling year. Samples were processed in a glove box under an argon atmosphere. The samples for POC-Out concentration as well as for ^13^C-POC-Out and ^14^C-POC-Out analyses were filtered (combusted GF/F) and acid-treated^26^. From every ^13^C-POC-Out sample one defined piece of filter was separated. The weight of pieces was proportional to the POC yields at individual sampling days. The pieces were combined to a bulk sample. The same was applied to the ^14^C-POC-Out samples. These time-integrating samples characterize the ^13^C and ^14^C of the POC exported from the reservoirs via the near spillover (POC-Out). Glassware used for isotope analysis was rinsed twice with 0.1 N HCl and baked at 500 °C for four hours.

From the jar containing the suspended sediment of the first core we collected gas from the headspace using a syringe after repeated cycles of shaking and sediment settling. We did not acidify the sediment samples to avoid a dissolution of carbonates such as calcite precipitates. The gas was directly injected into the gas chromatograph to measure the concentrations of CH_4_-Sed and of CO_2_-Sed (see Isotope and water chemistry analyses below). In addition, depending on concentrations 4–12 mL gas was transferred into UHP nitrogen flushed vials (Exetainer 12 mL, Labco, Lampeter, UK) crimped with septum caps (butyl/PTFE) for later analysis of ^2^H-CH_4_-Sed, ^13^C-CH_4_-Sed and ^13^C-CO_2_-Sed (see below). To collect the methane for ^14^C analysis, we used two sharpened metal tubes (1 mm inner diameter) that were guided to the two septa of the lid (see above). One tube only reached the gas headspace, the second was moved into the liquid layer near to the bottom of the jar. The gas of the headspace was flushed by a stream of UHP nitrogen and the methane was purified with the help of a liquid nitrogen trap removing CO_2_ and water vapor and then oxidized at 870 °C under continuous oxygen supply. The produced CO_2_ was reduced with H_2_ at 600 °C to graphite^32^ for accelerator mass spectrometry (AMS). Finally, a portion of the sediment from the first core was centrifugated (3750 rpm, 20 min) and dried at 105 °C to estimate the bulk density.

The sediment of the second core was flushed with UHP nitrogen using a septum lid and the stripped CO_2_ was precipitated in a barium hydroxide solution^31^ for ^14^C measurement by AMS. We dried the sediment for estimation of the bulk density.

The sediment sampled by the third core was dried at 60 °C and checked for roots and impurities with the help of a dissecting microscope. Subsamples were weighed and treated with HCl (37%) to remove inorganic carbon, dried again at 60 °C for 4 hours, crimped in silver boats and stored in a desiccator until POC-Sed analysis. Organic carbon for isotope analysis was extracted by the acid-base-acid method^33^. Subsamples were combusted at 900 °C in presence of CuO and a silver catalyst and graphitized for AMS. Aliquots were shipped in tin boats for ^13^C analysis by mass spectrometry.

### Isotope and water chemistry analyses, calculations

The TIC-Sur was quantified using a Dimatoc 2000 analyser (Analysentechnik, Essen, Germany). For POC-Out and POC-Sed a Vario EL analyser was used (Elementar, Hanau, Germany). We related the POC-Sed values to the volume of the sediment using the measured bulk density of the subsamples (see below). The CH_4_-Sed and CO_2_-Sed concentrations were analysed by headspace gas chromatography (SRI 8610 with flame ionisation detector and methaniser, SRI Instruments, Torrance, USA)^34^.

Radiocarbon was analysed by AMS (3 MV HVEE Tandetron 4130)^32^. Values of ∆^14^C express the carbon isotope ratio as deviations in per mil (‰) from the oxalic acid II standard (SRM 4990 C). They were corrected for process and instrument blanks and for isotope fractionation^35^ by the AMS system. We calculated the conventional radiocarbon age (CRA) relative to the year 1950 AD as year 0 BP and on the basis of a ^14^C half-life of 5568 years^35^.

Gas isotope samples (^2^H-CH_4_-Sed, ^13^C-CH_4_-Sed and ^13^C-CO_2_-Sed) were sent in duplicates to the Stable Isotope Facility at the University of California, Davis, USA. Analyses were performed using a Thermo Scientific GasBenchII plus PreCon(centration) device coupled to a Thermo Scientific Delta V Plus isotope-ratio mass spectrometer (IRMS)^36^. The ^2^H abundances were expressed with respect to V-SMOW (Vienna Standard Mean Ocean Water). The ^13^C values were expressed relative to standard Vienna PeeDee Belemnite. The results were not corrected for headspace fractionation as the gases were thoroughly transferred to the headspace in the saturated sodium chloride solution (see above). Furthermore, the ^13^C-CO_2_-Sed were not corrected for fractionation within the bicarbonate system. Depending on pH, the CO_2_ can be 0–9‰ more depleted than the dissolved inorganic carbon^37^. This introduces a small uncertainty in our ^13^C-CO_2_-Sed estimates, which does not affect our conclusions. The ^13^C abundances of TIC-Sur as well as of POC-Out were analysed by MS (Thermo Scientific Delta V IRMS) at the Colorado Plateau Stable Isotope Laboratory, Flagstaff, USA. Error ranges of analytical methods are given in Table S1.

We also measured the bulk density of the first and second sediment core. We calculated the water content from the difference of the net weight of the sample and the dry weight (DW). The volume of the sediment was estimated as water content minus 0.5. DW assuming a specific density of the solid fraction of 2.0 g mL^−1^.

## Results and Discussion

### Isotopic composition of methane and of carbon sources

**Figure 1:**
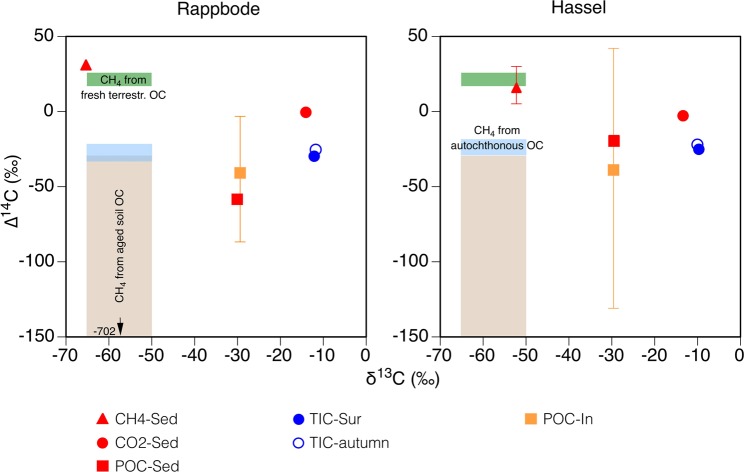
Isotopic composition of methane and of potential carbon sources. The ∆^14^C-CH_4_-Sed values show the means ± SD of three samples from Hassel reservoir and one sample ± analytical error from Rappbode reservoir. ‘POC-In’ symbols represent means and ranges of five to seven ^14^C samplings. Other ranges or analytical errors were smaller than symbol sizes. For further information see Table 3 and Table S1. The coloured boxes show the possible isotopic ranges of methane produced by biodegradation of different OC sources. We highlighted potential sources for which radiocarbon analyses were available from the sampling year. That is, fresh terrestrial OC such as tree needles (∆^14^C-POC-Needles 17‰ to 26‰, dark green) as well as aged soil OC (∆^14^C-POC-Soil −29‰ to −702‰, brown)^26^. With respect to autochthonous OC produced by internal photosynthesis, the possible ∆^14^C-CH_4_ values correspond to the ∆^14^C-TIC, i.e. to the ∆^14^C of TIC in surface samples integrated over the sampling year (∆^14^C-TIC-Sur, blue) as well as to the TIC in the autumn samples (∆^14^C-TIC-autumn, blue). Note that the ∆^14^C values of the source carbon (e.g. soil OC, autochthonous OC) can be transferred to the product (methane) as ∆^14^C values are corrected for fractionation by convention^35^. However, δ^13^C values are not corrected. The predicted ranges (coloured boxes) represent typical δ^13^C values for methane from acetate fermentation in freshwater habitats^59^. There is some overlap in ∆^14^C of methane deriving from aged terrestrial OC and from autochthonous OC in Rappbode but not in Hassel reservoir. Figure S1 provides a black and white version.

The methane in the sediments of both reservoirs was of modern origin (∆^14^C-CH_4_-Sed > 0‰, Fig. 1, Table 3). This means that it included ^14^C-enriched carbon released by nuclear testing after 1950. The samples from Hassel reservoir yielded ∆^14^C values of 5 ± 3‰ and 13 ± 3‰ from two cores taken at the deepest station as well as of 30 ± 3‰ from the core of the shallow site (single measurements ± analytical errors). The Rappbode reservoir sample contained methane with a ∆^14^C of 31 ± 3‰.

**Table 3:** Size and Isotopic composition of carbon pools. For description of parameters see Table 2.

	Rappbode reservoir			Hassel reservoir		
	Concentration	∆^14^C (‰)	δ^13^C (‰)	Concentration	∆^14^C (‰)	δ^13^C (‰)
***Stream inflow (reservoir import)***						
POC-In^*a^	0.5 ± 0.3 mg C L^−1^	−86.8 to –3.2	−30.6 to –28.4	0.8 ± 0.4 mg C L^−1^	−131 to 42	−30.2 to –29.2
***Outflow (reservoir export)***						
POC-Out^*b^	0.8 ± 0.3 mg C L^−1^	−41.8 ± 7.2	n.d.	1.2 ± 0.7 mg C L^−1^	−43.1 ± 6.7	n.d.
***Surface layer of reservoir***						
TIC-Sur^*b^	8.6 ± 2.7 mg C L^−1^	−29.8 ± 3.8	−12.1 ± 0.0	9.6 ± 3.1 mg C L^−1^	−25.1 ± 1.7	−9.7 ± 0.0
TIC-autumn^*c^	11.3 mg C L^−1^	−25.4 ± 3.5	−11.8 ± 0.0	13.3 mg C L^−1^	−22.0 ± 4.7	−10.1 ± 0.2
***Sediment of reservoir*** ^*d^						
Water content	961 g dm^3^			969 g dm^3^		
Bulk density	78 g dm^3^			62 g dm^3^		
CO_2_-Sed	0.3 mg C dm^3^	−0.5 ± 3.7	−14.1 ± 0.1	1.0 mg C dm^3^	−2.8 ± 3.7	−13.4 ± 0.1
POC-Sed	6017 mg C dm^3^	−58.4 ± 4.4	−30.1 ± 0.0	6646 mg C dm^3^	−19.7 ± 3.9	−29.5 ± 0.0
CH_4_-Sed	6.9 mg C dm^3^	31.1 ± 2.9^*e^	−65.3 ± 0.5	1.6 mg C dm^3^	5.1 to 30.0^*e^	−52.3 ± 0.8

^*a^Concentrations: means ± SD. Isotopes: min - max values of 4–7 samplings.

^*b^Concentrations: means ± SD. Isotopes: means ± 0.5 ranges of duplicate measurements.

^*c^∆^14^C: single measurements ± analytical errors. δ^13^C: duplicate measurements of one sample (means ± 0.5 ranges).

^*d^Individual samples. ∆^14^C: single measurements ± analytical errors. δ^13^C: duplicate measurements (means ± 0.5 ranges). Carbon concentrations are expressed relative to total sediment volume.

^*e^Individual isotope sample from Rappbode reservoir, range of three samples from Hassel reservoir (mean 16.0‰).

To assess the origin of OC supporting methane production it is crucial to constrain the isotopic signatures of potential carbon sources. The photosynthetically fixed carbon in all algal species carries a uniform ∆^14^C signature, which is the ∆^14^C value of the CO_2_ in the photic zone. According to standard practice ∆^14^C values are corrected for fractionation by convention^35^, i.e. for fractionation within the system of inorganic carbon species or during photosynthesis. Therefore, the ∆^14^C-TIC-Sur constrains the radiocarbon signal of autochthonous OC and of methane deriving from this source (blue boxes in Fig. 1).

To estimate the ∆^14^C of CO_2_ available for photosynthesis, we used time-integrating samples from the surface layers of the reservoirs covering the sampling year (∆^14^C-TIC-Sur, Methods). In both reservoirs, they contained radiocarbon depleted TIC (∆^14^C −30 ± 4‰ and −25 ± 2‰, means ± 0.5 ranges of duplicate measurements, Rappbode and Hassel reservoirs, respectively; Fig. 1, Table 3). These results agree well with those from individual samples of surface layers measured at the end of summer stratification (∆^14^C-TIC-autumn, −25‰ and −22‰).

In contrast to autochthonous OC produced by photosynthesis, terrestrial OC constitutes a mix of compounds derived from various terrestrial sources (e.g. fresh leaves, aged soil OC) with varying ages and hence radiocarbon contents. The ∆^14^C of compounds used for methane production can differ from the average ∆^14^C of all compounds of a bulk sample. The individual POC inflow samples (POC-In) covered a wide range in ∆^14^C between −3‰ and −94‰ in Rappbode reservoir as well as between 42‰ and −131‰ in Hassel reservoir (Fig. 1). The most negative ∆^14^C here corresponds to a CRA of 1067 years BP. Previous studies had shown that during the stratified period at our study sites the diffusive flux of DOC was always directed from the sediment into the water^29^. Therefore, we do not consider terrestrial DOC a relevant source of methane, although more DOC than POC was delivered by the inflows^26^ (Table 1).

The ∆^14^C-CH_4_-Sed in both reservoirs was near to or within the range of ∆^14^C of recently fixed terrestrial OC. In February 2013 we picked two samples of fresh green needles (POC-Needles, Table 2) from spruce trees. The measured ∆^14^C values of 23 ± 3‰ and 20 ± 3‰ were close to those of the methane (Fig. 1). This points to fresh terrestrial plant biomass as the methane source. Freshly fixed OC can be delivered to the stream by overland flow eroding the uppermost soil layer^38^ as well as by litterfall of canopy trees^39^.

Although the radiocarbon content of the methane nearly matched that of fresh vegetation, we need to consider the possibility that terrestrial OC with more positive ∆^14^C values was involved into methane production. This OC could be some years to decades older than that of the sampling year but will be more ^14^C enriched. The reason is that due to nuclear tests after 1950, atmospheric ∆^14^C-CO_2_ summer values almost doubled and then decreased from nearly 1000‰ in 1964 to 87‰ in 2000 and to 31‰ in 2012^40,41^. In contrast, terrestrial plant biomass produced before 1950 is depleted in ^14^C (Fig. 1). More positive ∆^14^C values of contemporary OC could be balanced with supplies of ^14^C-negative sources such as aged terrestrial OC. In our catchments, aged terrestrial OC (POC-Soil) presumably derived from erosion of the stream bank. Its components spanned a gradient in ∆^14^C ranging potentially from moderately negative values such as −29‰ as in POC-Soil at 8 cm depth to −702‰ as in POC-Soil at 81 cm depth of the catchment soil (Fig. 1). For the most depleted soil OC we calculated a CRA of 9650 years corresponding to its formation after Pleistocene glaciation. Out of the three ∆^14^C-CH_4_-Sed measurements of the Hassel reservoir, two values (5‰, 13‰) were below the ∆^14^C of fresh OC (31‰, see above). Positive ∆^14^C values below that of fresh OC existed only transiently in the atmosphere. Therefore, a ^14^C-depleted source with pre-bomb ^14^C must have contributed to methane production. We conclude that contemporary terrestrial vegetation was the predominant carbon source, supported by ^14^C-depleted OC of autochthonous or of terrestrial origin.

From the methane we also obtained measurements of stable carbon and hydrogen isotopes. The δ^13^C-CH_4_-Sed and the δ^2^H-CH_4_-Sed values amounted to −65‰ and −52‰ (Fig. 1) as well as to −295‰ and −313‰ (not shown) in Rappbode and Hassel reservoirs, respectively. In combination they suggest that the methane production was based on acetate fermentation rather than CO_2_ reduction^42^. However, for a precise estimate we need to know the δ^13^C of the acetate methyl group and the fractionation factors of involved methanogenic pathways^43^. If hydrogenotrophic methanogenesis was taking place, the ^14^C signature of the methane would be constrained by the ^14^C signature of CO_2_. The ∆^14^C-CO_2_-Sed values were ∼0‰ and relatively near to those of the fresh terrestrial OC, which means that the CO_2_ was likely produced from the mineralisation of terrestrial OC. If CO_2_ was actually a methane source, fresh terrestrial OC must have been also a significant basis of methane formation. This would not change our conclusions.

We also measured ^14^C-enriched methane in two other reservoirs (Santa Fe ∆^14^C-CH_4_-Sed 82‰, Bautzen 9‰, not shown). The TIC-Sur was also modern (27‰ and 15‰, respectively). The methane sources of the eutrophic Bautzen reservoir cannot be distinguished as the ∆^14^C-CH_4_-Sed was near to the ∆^14^C of autochthonous OC (TIC-Sur) and near to the ∆^14^C of fresh terrestrial OC. The values for the oligotrophic Santa Fe reservoir show that the methane contained terrestrial OC that was more enriched than the recently fixed fraction. Values equal to or higher than 82‰ occurred in atmospheric CO_2_ in 2001 and before. The significance of terrestrial OC is consistent with the characteristics of the very small Santa Fe reservoir (7 ha) which is closely surrounded by deciduous forest. This is in line with our conclusion that the methane production was based on contemporary terrestrial biomass with supplements of aged terrestrial or autochthonous sources.

### Terrestrial vs. autochthonous OC supply and methane production

Annually the reservoirs received nearly equal amounts of terrestrial POC via the inflowing streams (POC-In, Table 1). However, the phytoplankton biomass was 2.5-fold higher and the photosynthetic OC production was 2.8-fold higher in Hassel reservoir than in Rappbode reservoir (Table 1). The biomass of diatoms – algae which efficiently transport OC to the sediment – was also higher in Hassel reservoir than in Rappbode reservoir (2.6 and 1.9 g C m^2^, respectively). Hence, terrestrial POC was more significant in Rappbode reservoir where it contributed 43% to the overall OC supply compared to 20% in Hassel reservoir.

The production of methane in the sediment can be estimated from its accumulation rate in the anoxic hypolimnion. Previous research had shown that the production of methane in the sediment of both reservoirs was equal to the flux of methane from the sediment into anoxic water where it accumulated during summer^34^. Seasonal flux measurements using floating chambers showed that ebullition of methane can be excluded (Table S2). Methane production rates in the sediments of both reservoirs were similar, 6 and 8 g C m^−2^ yr^−1^ in Rappbode and Hassel respectively (Table 1). These rates can be extrapolated to shallower areas were methane production in the anoxic sediment was probably similar, although methane did not reach the water column because it was oxidized at the sediment surface^44^. By comparing the concentration of methane in the sediment with the methane production rates we can estimate that all methane in the sediment was produced within one year prior to sampling. The production of methane was low relative to the OC production in the reservoirs and to the import of OC from catchments (Table 1).

**Figure 2:**
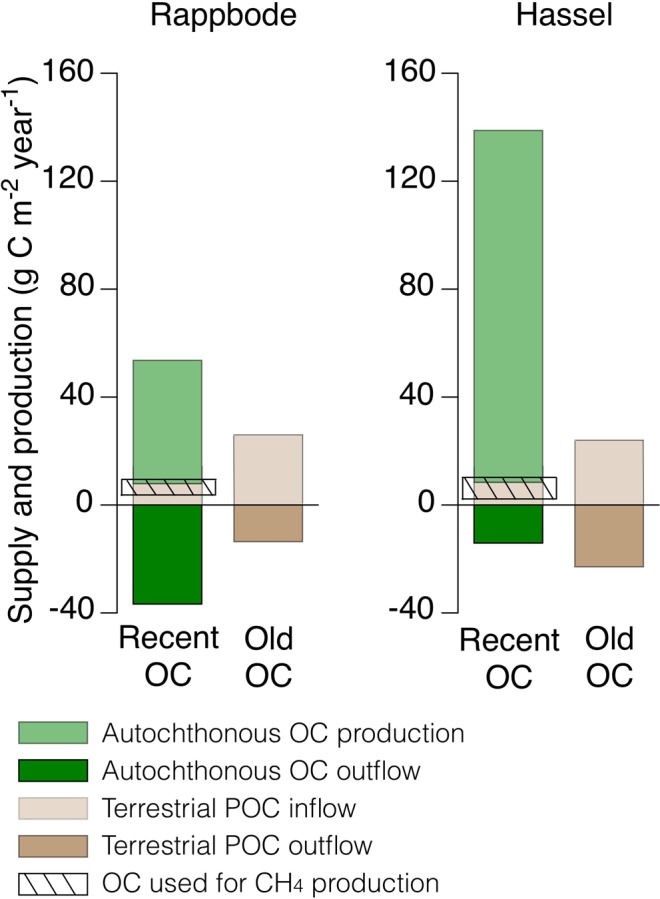
The carbon sources of methane. Recent OC comprised autochthonous biomass produced during the sampling year (Table 1) as well as fresh terrestrial OC (∆^14^C-POC-Needles 17‰ to 26‰, see text). Old OC was of terrestrial origin (POC-Soil). The carbon fluxes and isotope ratios were calculated by a mass budget (Table S3). The hatched areas indicate the OC used for methane production as derived from the discussion.

Although more OC was provided by internal photosynthesis than by stream POC, the methane production was based predominantly on contemporary terrestrial OC. This source represents only a small fraction of the OC theoretically available for methanogenesis (Fig. 2). Unlike the majority of the terrestrial POC, the autochthonous OC may be readily available^8,39,45–47^ and preferentially decomposed under aerobic conditions or by other energetically more efficient pathways than methanogenesis such as denitrification, iron und sulphate reduction. This can take place already in the water column and at the sediment-water interface^48^. Mass balance calculations using ^13^C revealed that the degradation of autochthonous OC contributed to the TIC pool in the hypolimnion of both reservoirs^49^. This supports the hypothesis that methanogenesis, as the terminal mineralization step, is faced with the more refractory leftovers of the other respiratory processes. This argumentation is in line with results from radiocarbon measurements from a stream draining peatland. The authors concluded that most of the younger CO_2_ was produced from the relatively rapid aerobic mineralization of organic matter, whereas methane production was restricted to older layers^50^.

We found that OC originating from contemporary terrestrial vegetation was the predominant source of methane in the sediments of our reservoirs. This agrees with results of a study conducted in bogs and fens of northern Minnesota. The authors emphasized the similarity of the ∆^14^C values of contemporaneous atmospheric CO_2_ and emitted methane^20^. The methane production in our two reservoirs was low, but in a range typical for temperate reservoirs^16^. Between 18% and 25% of stream POC supplies were sufficient to sustain the measured methane production rates in the Rappbode and Hassel reservoirs, respectively (Table 1). The predominant use of a comparatively small and young fraction of the terrestrial POC might be related to the low methane production and interpreted as a characteristic of our systems. Rising emissions, however, do not imply that a larger fraction of terrestrial POC is required to maintain methanogenesis. This is exemplified with the temperate hydropower reservoir Lake Wohlen, where only 3% of riverine POC inputs could sustain extreme emissions that were 15 to 20 times higher than in our reservoirs^18,51,52^. Secondly, soil organic carbon and plant material are significant sources of methane production after flooding^15^. If it takes about 40 years until continued inputs from inflowing rivers and internal photosynthesis may become the main sources^16^, flooded soil is not a relevant carbon source for methanogenesis in our reservoirs.

Our results seem to contradict recent studies which show that methane emissions increase with productivity of lakes and reservoirs^10,53^. The large influence of allochthonous carbon in our study can be explained by the small size of the reservoirs investigated here. Our results can refer to the river-reservoir transition zone of larger reservoirs or run-off-the-river dams where large portions of the suspended matter are initially deposited and which are emission hotspots^54–56^. In reservoirs larger than in this study, internal photosynthesis may become a more significant source. It has been shown that sedimentation areas at river inflows into lakes are rich in terrestrial material^48^ and exhibit high rates of methanogenesis^57^. Our results suggest that terrestrial carbon is a major driver of such methanogenesis hotspots.

This is the first study of radiocarbon in methane together with its potential sources in a reservoir. The results demonstrate that the potential sources of methane must be isotopically well characterized, i.e. a presence of radiocarbon-enriched methane alone would not sufficiently support the conclusion that its production was based on internal photosynthesis. Although more OC was supplied by internal photosynthesis than by stream POC, the methane production was fuelled by terrestrial OC, predominantly of contemporary origin.

## Supplementary information

Table S1, Table S2 and Figure S1

Table S3 (Supplementary Dataset)
