# Supplementary material for: Terrestrial Vegetation Drives Methane Production in the Sediments of two German Reservoirs
Source: Sci Rep. 2019 Nov 4;9:15944. doi: 10.1038/s41598-019-52288-1 (PMC6828770; doi:10.1038/s41598-019-52288-1)
Supplement: Supplementary file 1 — Table S1, Table S2 and Figure S1 [file 41598_2019_52288_MOESM1_ESM.docx]

**Supplementary Information to " TERRESTRIAL VEGETATION DRIVES METHANE PRODUCTION IN THE SEDIMENTS OF TWO GERMAN RESERVOIRS"**

by Jörg Tittel^1^, Matthias Hüls^2^, and Matthias Koschorreck^1^

^1^Helmholtz Centre for Environmental Research - UFZ, Department Lake Research, Brückstrasse 3a, D-39114 Magdeburg, Germany.

^2^Leibniz-Laboratory for Radiometric Dating and Stable Isotope Research, Max-Eyth-Str. 11-13, D-24118 Kiel, Germany.

Corresponding author: Jörg Tittel (joerg.tittel@ufz.de), ORCID 0000-0003-1151-1909

**Content:**

Table S1: Errors of replicate measurements of samples or analytical standards

Table S2: Measurement of the methane fluxes from the reservoirs into the atmosphere

Legend Figure S1: Isotopic composition of methane and of potential carbon sources

Figure S1

**Table S1**: Errors of replicate measurements of samples or analytical standards

| Parameter | Analyzer | SD or range | |
| --- | --- | --- | --- |
|  |  | Samples | Standard |
|  |  |  |  |
| TIC-Sur | Dimatoc 2000 | 0.07 mg C L^-1 *^ | 0.16 mg C L^-1 #^ |
| POC-Sur | Vario EL Element Analyzer | 0.02 mg C ^**^ | 0.03 mg C  ^#^ |
| CH_4_-Sed | SRI GC8610 | 9.6 10^-4^ mg C dm^-3 *^ | 3.2 10^-5^ mg C dm^-3 #^ |
| CO_2_-Sed | SRI GC8610 | 1.1 10^-2^ mg C dm^-3 *^ | 1.2 10^-3^ mg C dm^-3 #^ |
| ^14^C (all samples) | HVEE Tandetron 4130 AMS System | 3 - 4 ‰^*^ | 3 - 4 ‰^#^ |
| ^2^H-CH_4_-Sed | Thermo Scientific Delta V Plus IRMS | 0.87 ‰^*^ | 1.53 ‰^#^ |
| ^13^C-CH_4_-Sed | Thermo Scientific Delta V Plus IRMS | 0.68 ‰^*^ | 0.15 ‰^#^ |
| ^13^C-CO_2_-Sed | Thermo Scientific Delta V Plus IRMS | 0.10 ‰^*^ | 0.02 ‰^#^ |
| ^13^C-TIC-Sur | Thermo Scientific Delta V IRMS | 0.20 ‰^**^ | 0.04 ‰^#^ |
| ^13^C-POC-Sur | Thermo Scientific Delta V IRMS | 0.20 ‰^**^ | 0.04 ‰^#^ |

^*^ - standard deviation of three or more measurements of parallel samples

^**^ - difference between two measurements of one sample

# - difference between measured and known concentration of an analytical standard

**Table S2**: Measurement of the methane fluxes from the reservoirs into the atmosphere.

Fluxes are in mmol m^-2^ d^-1^. Area [%] denotes the part of the total reservoir surface for which the sampling site was representative for, distance is the distance to the dam ^1^. Days denote the time span for which the particular sampling was considered representative. Bold numbers indicate ebullition.

| Site code | Distance [m] | Area [%] | 21.04.2015 | 16.07.2015 | 28.10.2015 | 25.11.2015 |
| --- | --- | --- | --- | --- | --- | --- |
|  |  |  |  |  |  |  |
| Hassel Dam | |  |  |  |  |  |
| YH3 | 61 | 23 | 0.11 | 0.23 | 0.00 | 0.72 |
| YH4 | 283 | 17 | 0.00 | 0.15 | 0.00 | 0.42 |
| YHE | 715 | 20 | 0.00 | 0.00 | 0.00 | 0.00 |
| YH5 | 1010 | 15 | 0.00 | 0.43 | 0.00 | 0.00 |
| YHF | 1200 | 9 | 0.00 | 0.56 | 0.00 | **0.93** |
| YHG | 1480 | 17 | 0.12 | **0.66** | **2.44** | 1.16 |
| mean |  |  | 0.05 | 0.30 | 0.41 | 0.51 |
| days |  |  | 91 | 91 | 60 | 60 |
|  |  |  |  |  |  |  |
| Hassel Dam | |  | [mmol m^-2^ d^-1^] | | [g C m^-2^ y^-1^] | |
| mean annual flux | |  | 0.24 |  | 1.04 |  |
|  |  |  |  |  |  |  |
| Rappbode Dam | |  |  |  |  |  |
| Site code | Distance [m] | Area [%] | 20.04.2015 | 15.07.2015 | 27.10.2015 | 24.11.2015 |
| YR3 | 35 | 38 | 0.32 | 0.21 | 0.00 | 0.65 |
| YRM | 600 | 40 | 0.54 | 0.35 | 0.00 | 0.51 |
| YRH | 1050 | 7 | 0.50 | 0.50 | 0.00 | 0.26 |
| YRI | 1300 | 4 | 0.74 | **18.53** | **2.32** | 0.33 |
| YRJ | 1500 | 12 | 0.65 | 1.24 | 0.00 | 0.42 |
| mean |  |  | 0.48 | 1.14 | 0.09 | 0.53 |
| days |  |  | 91 | 91 | 60 | 60 |
|  |  |  |  |  |  |  |
| Rappbode Dam | |  | [mmol m^-2^ d^-1^] | | [g C m^-2^ y^-1^] | |
| mean annual flux | |  | 0.50 |  | 2.21 |  |

The flux of CH_4_ between the reservoirs and the atmosphere was measured in 2015 using floating chambers. To cover seasonal dynamics at each reservoir 4 samplings were performed. To cover spatial heterogeneity lateral transects with 6 sites in Hassel and 5 sites in Rappbode dam were sampled.

We used a floating chamber connected to a mobile FTIR analyzer (GASMET DX4000, Temet Instruments, Finland) ^2^. The CH_4_ flux was calculated from the linear increase of CH_4_ in the chamber during 3-5 min measurements. Ebullition was only rarely observed. In that case the flux was calculated from the difference between start and end concentration during flux measurements.

For each sampling date the mean CH_4_ flux for the whole reservoir was calculated as the area weighted mean of the different sampling sites ^1^. To compute the annual mean flux the 4 samplings were assumed representative for 3 months (spring and summer) or 2 month (autumn). We assumed two months of ice cover and zero flux.

The mean annual CH_4_ flux to the atmosphere was 1.04 and 2.21 g C m^-2^ y^-1^ for Hassel and Rappbode respectively (Table S1). Highest fluxes were observed in the inflow area and close to the dam. Ebullition was only detected near to the inflow.

References

1 Perz, A. *Longitudinal Heterogeneity in Two Pre-Reservoirs in the Harz Mountains (Germany)* master thesis, Swedish University of Agricultural Sciences, (2013).

2 Gomez-Gener, L. *et al.* Hot spots for carbon emissions from Mediterranean fluvial networks during summer drought. *Biogeochemistry* **125**, 409-426, doi:10.1007/s10533-015-0139-7 (2015).

**Figure S1**: Isotopic composition of methane and of potential carbon sources.

The ∆^14^C-CH_4_-Sed values show the mean ± SD of three samples in Hassel reservoir and one sample ± analytical error in Rappbode reservoir. 'POC-In' symbols represent means and ranges of five to seven ^14^C samplings. Other ranges or analytical errors were smaller than symbol sizes. For further information see Table 3 and Table S1. The boxes show the possible isotopic ranges of methane produced by biodegradation of different OC sources. We highlighted potential sources for which radiocarbon analyses were available from the sampling year. That is, fresh terrestrial OC such as tree needles (∆^14^C-POC-Needles 17 ‰ to 26 ‰, grey) as well as aged soil OC (∆^14^C-POC-Soil -29 ‰ to -702 ‰, hatched) ^1^. With respect to internal photosynthetic biomass (autochthonous OC), the possible ∆^14^C-CH_4_ values correspond to the ∆^14^C-TIC, i.e. to the ∆^14^C of TIC in surface samples integrated over the sampling year (∆^14^C-TIC-Sur, transparent) as well as to the TIC in the autumn samples (∆^14^C-TIC-autumn, transparent). Note that the ∆^14^C values of the source carbon (e.g. soil OC, autochthonous OC) can be transferred to the product (methane) as ∆^14^C values are corrected for fractionation by convention ^2^. However, δ^13^C values are not corrected. The predicted ranges (boxes) represent typical δ^13^C values for methane from acetate fermentation in freshwater habitats ^3^. There is some overlap in ∆^14^C of methane deriving from aged terrestrial OC and from autochthonous OC in Rappbode but not in Hassel reservoir.

References

1 Tittel, J., Müller, C., Schultze, M., Musolff, A. & Knöller, K. Fluvial radiocarbon and its temporal variability during contrasting hydrological conditions. *Biogeochemistry*, 1-13, doi:10.1007/s10533-015-0137-9 (2015).

2 Stuiver, M. & Polach, H. A. Reporting of ^14^C Data - Discussion. *Radiocarbon* **19**, 355-363 (1977).

3 Whiticar, M. J., Faber, E. & Schoell, M. Biogenic methane formation in marine and freshwater environments: CO_2_ reduction vs. acetate fermentation - Isotope evidence. *Geochim.Cosmochim, Acta.* **50**, 693-709 (1986).


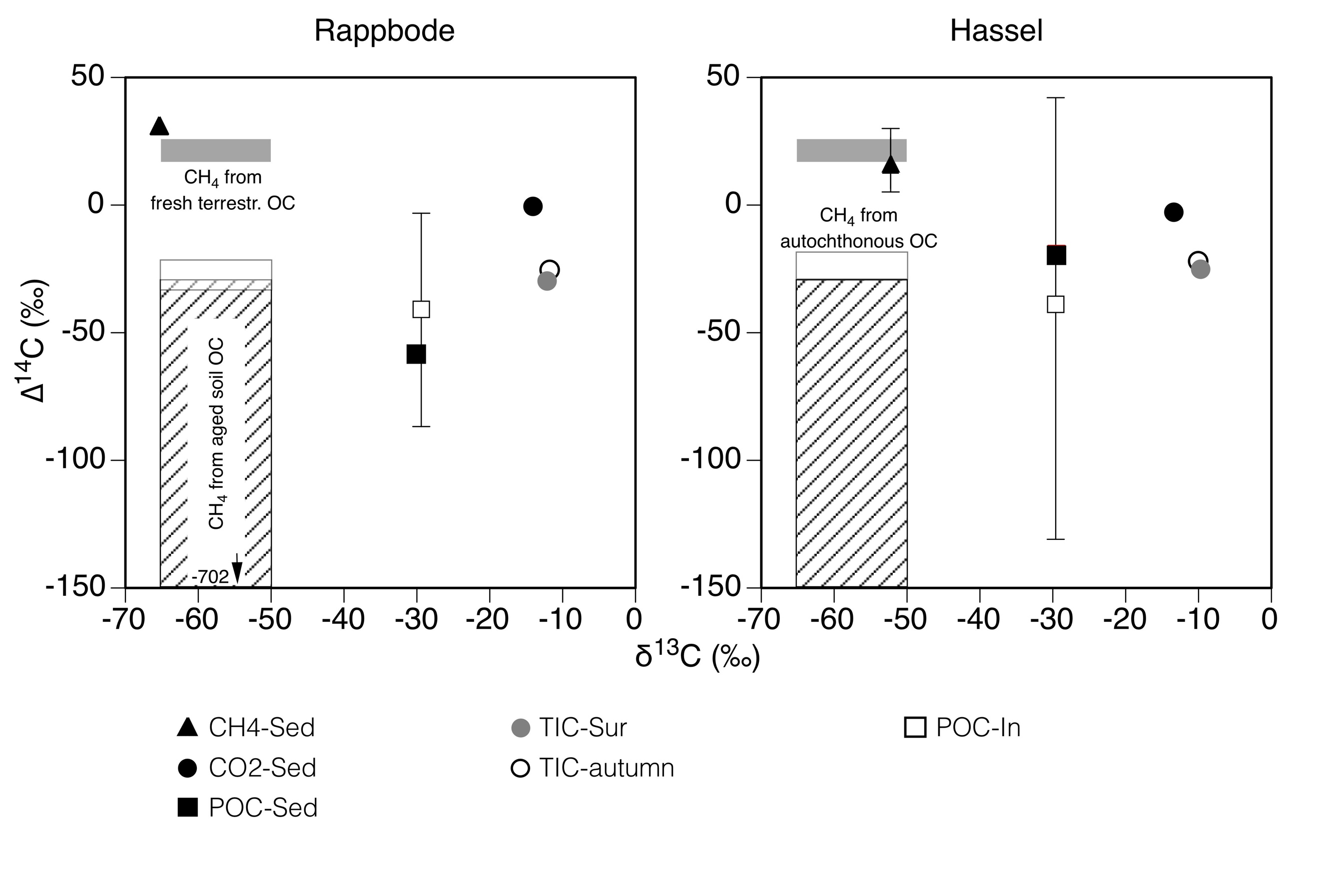


**Figure S1**
